# Macrodystrophia Lipomatosa Involving Both Median and Tibial Nerves: A Two-Case Series

**DOI:** 10.7759/cureus.81552

**Published:** 2025-03-31

**Authors:** Rishab T Ramesh, Koganti Venkata Sai Mani Deepak, Anupama Chandrasekharan, Veena M Joseph

**Affiliations:** 1 Department of Radiology, Sri Ramachandra Institute of Higher Education and Research, Chennai, IND; 2 Department of Radiology and Imaging, North Eastern Indira Gandhi Regional Institute of Health and Medical Sciences, Shillong, IND

**Keywords:** enlarged digits, fibrolipomatous hamartoma (flh), macrodystrophia lipomatosa, median nerve, tibial nerve

## Abstract

Macrodystrophia lipomatosa (MDL) is a rare congenital disorder marked by excessive proliferation of mesenchymal tissues, predominantly adipose tissue, which often manifests in the upper extremities (particularly the median nerve) and less frequently in the lower extremities (such as the tibial nerve). When MDL affects peripheral nerves, it is commonly associated with fibrolipomatous hamartoma (FLH), a benign fibrofatty overgrowth. The MRI is central to diagnosing MDL, as it provides critical visualization of pathological fat infiltration within the enlarged nerve. We present two cases illustrating the MRI characteristics of MDL in both the median and tibial nerves. Recognizing these hallmark radiological features is essential for accurate diagnosis, differentiation from other neuropathies, and informed management.

## Introduction

Macrodystrophia lipomatosa (MDL) is a rare congenital anomaly characterized by an overgrowth of mesenchymal elements, most prominently adipose tissue, that leads to disproportionate limb enlargement [1]. It often affects the upper extremities [2], especially along the median nerve distribution, but also presents in the lower limb (e.g., the tibial nerve), albeit less commonly. The MDL is frequently associated with fibrolipomatous hamartoma (FLH), a benign fibrofatty overgrowth within the nerve, contributing to the condition's clinical and radiologic features [2].

The MRI is the principal diagnostic tool, offering detailed visualization of adipose and fibrous tissue proliferation within the enlarged nerves [3]. This report details the MRI findings in two patients: one with median nerve involvement leading to digital overgrowth, and another with tibial nerve involvement presenting as an enlarged great toe. Understanding these characteristic MRI signs is critical for distinguishing MDL from other neuropathies with overlapping clinical presentations.

## Case presentation

Case one

**Figure 1:**
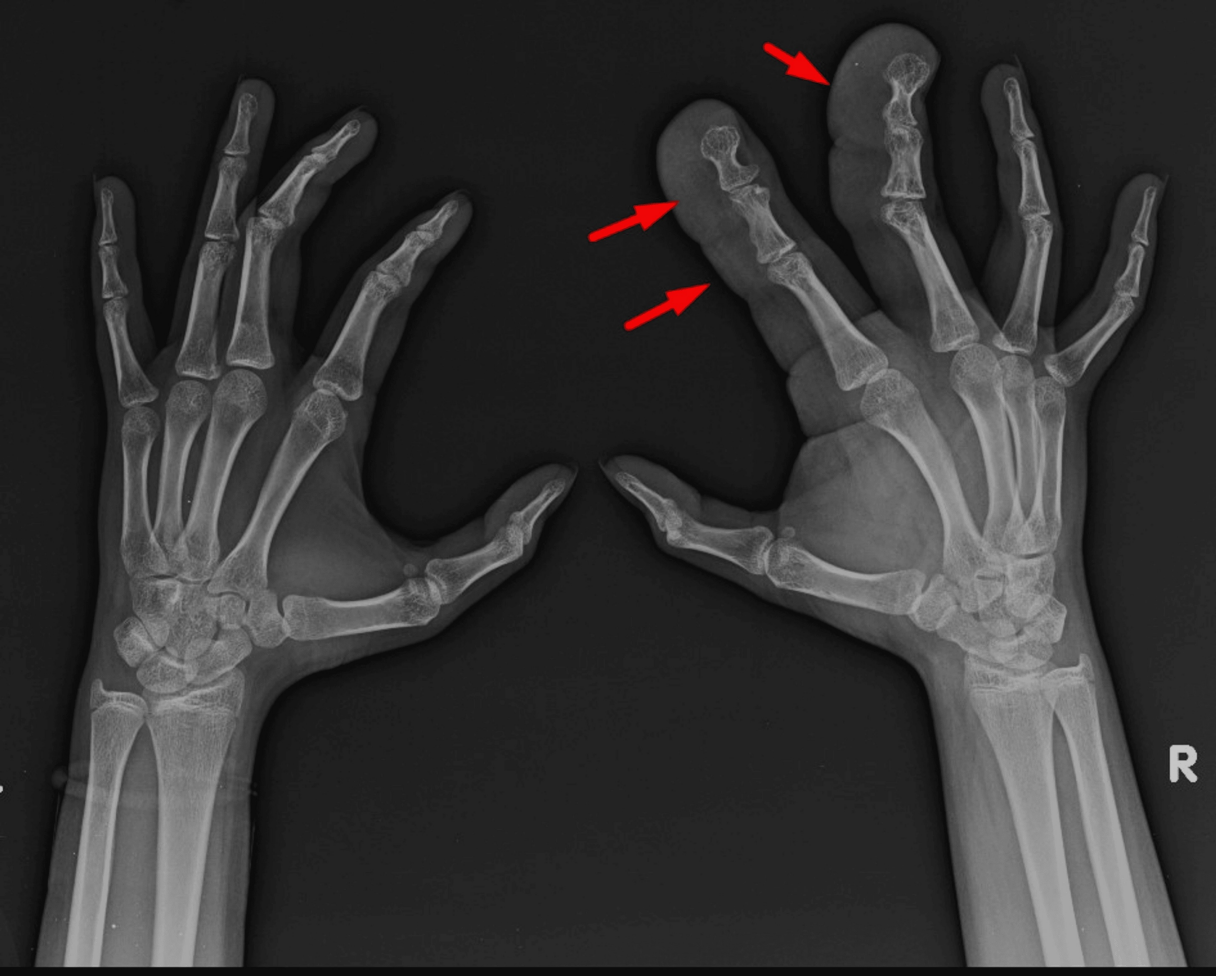
Radiograph of both hands The red arrows point at the diffuse enlargement of the second and third digits of the right hand with osseous hypertrophy in the form of broadening, elongation of phalanges with cortical thickening and adjacent soft tissue swelling.

A 13-year-old female complained of progressive enlargement of her right middle and index fingers since birth, without pain or movement restriction. She met all normal developmental milestones for her age and had no notable functional deficits. Radiography of the right wrist and hand showed diffuse enlargement of the second and third digits, osseous hypertrophy, cortical thickening, and associated soft tissue swelling (Figure 1).

**Figure 2:**
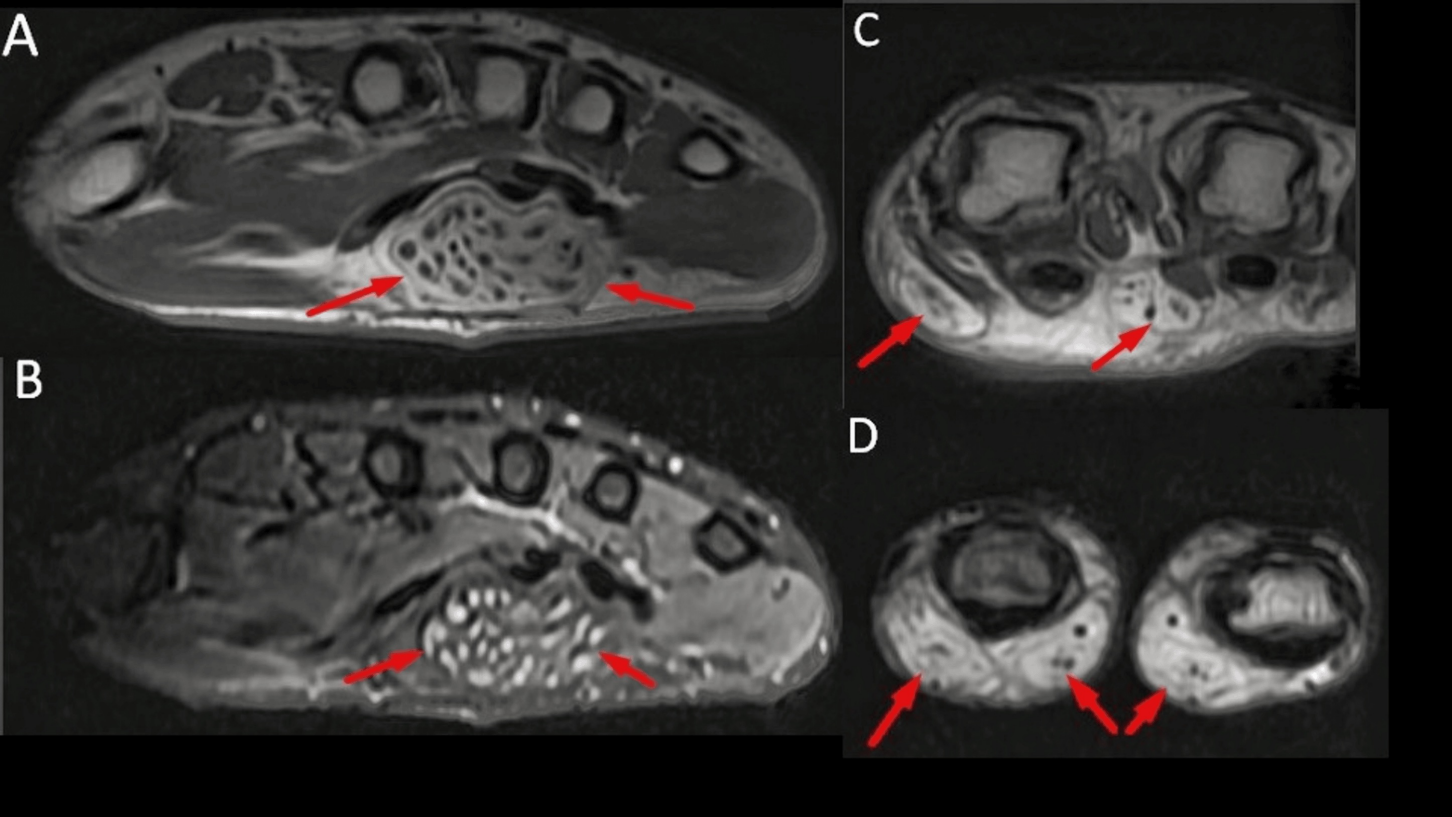
The MRI of the hand The T1, non-fat suppressed (A) and short tau inversion recovery (STIR) images (B) show the enlarged median nerve with fatty infiltration in the carpal tunnel and is seen causing indentation on the underlying flexor tendons and bulging of the overlying flexor retinaculum (C). The MRI T1, non-fat suppressed images (D) show thickened fascicles and surrounding lipomatous infiltration median nerve and its digital branches of the second and third digits.

The MRI demonstrated extensive subcutaneous fat with thin fibrous septations and fatty infiltration of the digital nerves for the second and third digits. The median nerve within the carpal tunnel appeared diffusely enlarged, with fatty strands indenting the underlying flexor tendons and bulging the flexor retinaculum. Thickened nerve fascicles surrounded by lipomatous tissue were visible in both the median nerve trunk and its digital branches (Figure 2). 

Given the patient's lack of pain or functional impairment, conservative management with physical therapy was advised to maintain hand function. Periodic X-ray and MRI monitoring was suggested in the follow-up visits to assess any changes in finger size or nerve compression.

Case two

**Figure 3:**
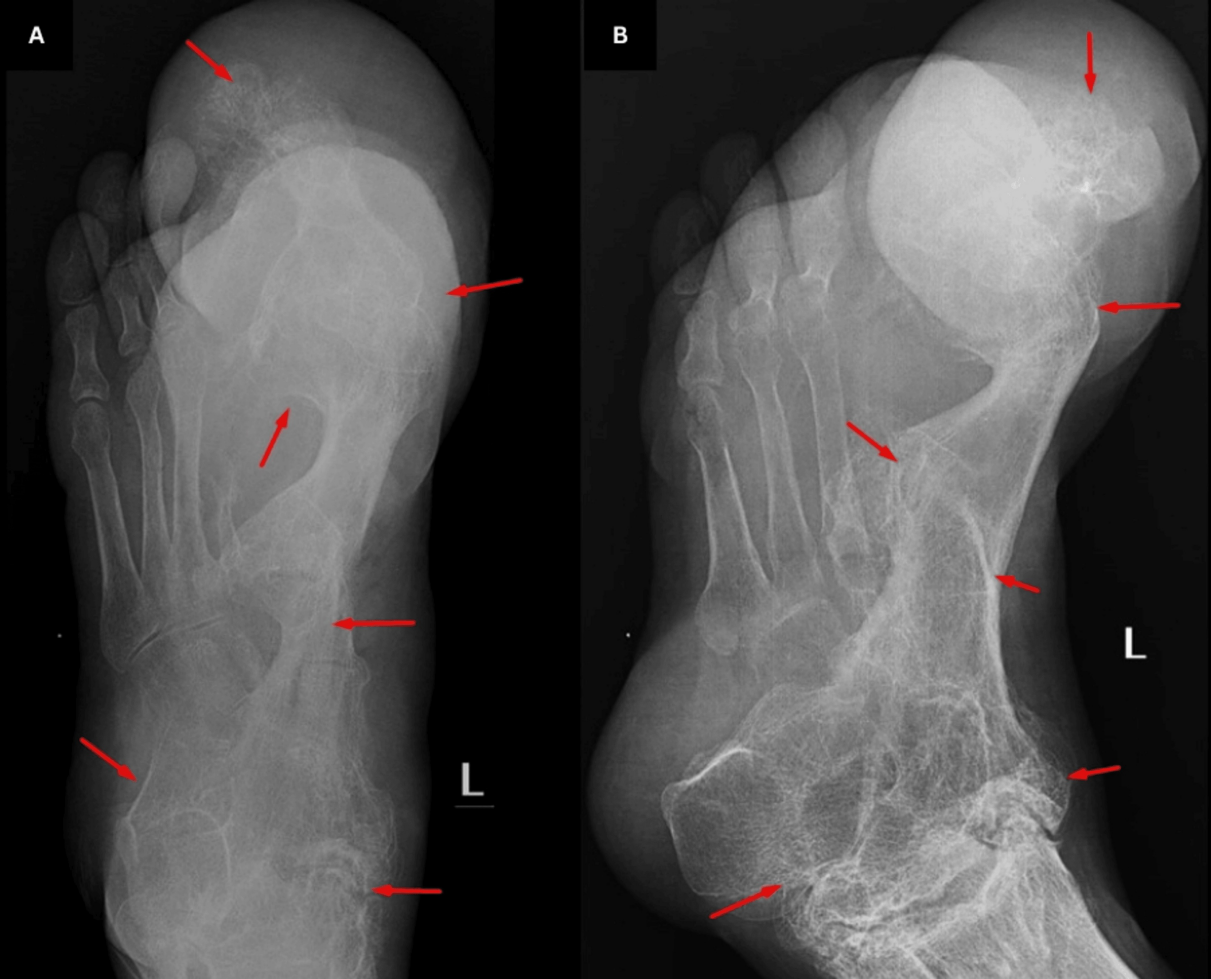
Radiograph of the foot A: Osseous hypertrophy of the first metatarsal bone and phalanges of the great toe with adjacent diffuse soft tissue enlargement; B: Bony ankylosis of all the tarsal bones, first tarso-metatarsal and interphalangeal joints of the great toe

A 39-year-old male with a prior diagnosis of plexiform neurofibromatosis presented with a progressively enlarging left great toe. He reported recent pain, ulceration, and discharge on the plantar aspect of the toe, but no limitation of motion. It was initially considered whether these new findings were related to his existing neurofibromatosis. However, the distinct imaging characteristics led to a different diagnosis. Radiography (anteroposterior, oblique views) of the foot revealed osseous hypertrophy of the first metatarsal and phalanges of the great toe with diffuse soft tissue enlargement. Bony ankylosis was noted in all tarsal bones, the first tarsometatarsal joint, and the interphalangeal joints of the great toe, most severely involving the medial tarsal bones (Figure 3). This bony ankylosis was a new finding compared to previous imaging.

**Figure 4:**
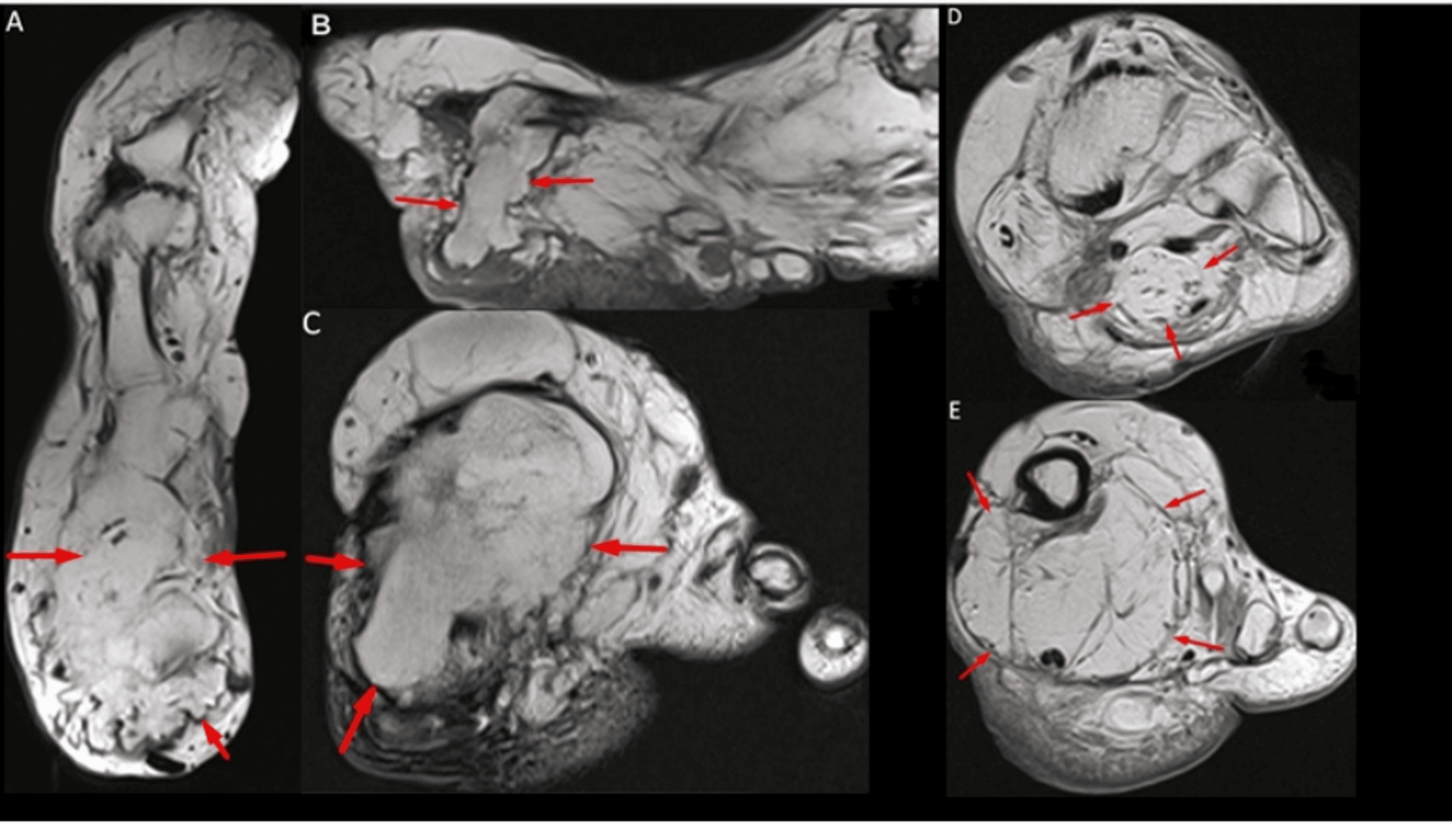
Multiplanar (T1 ,T2, non-fat suppressed images) MRI images of the foot The MRI shows a vertically oriented bony excrescence extending inferiorly from the head of the first metatarsal on the plantar aspect, resulting in plantar displacement of overlying soft tissues (A and B). This bony projection, along with the fat, is seen to compress and displace the flexor hallucis longus tendon inferolaterally (C). Image D shows the mild thickening of the fascicles and surrounding lipomatous infiltration tibial nerve and image E shows the fibromatous infiltration of the surrounding soft tissue.

The MRI (T1-weighted, non-fat suppressed, T2-weighted, and STIR sequences) showed a vertically oriented bony outgrowth extending inferiorly from the first metatarsal head, causing mild STIR hyperintensities and plantar displacement of the overlying soft tissues. This bony prominence, along with adjacent fat, compressed and displaced the flexor hallucis longus tendon inferolaterally (Figure 4). Additionally, there was mild fascicular thickening with fatty infiltration of the tibial nerve. The relationship between the tibial nerve involvement and the plantar symptoms is unclear.

While the MRI shows mild changes in the tibial nerve, the plantar pain, ulceration, and discharge are likely primarily related to the bony outgrowth and its mass effect on the surrounding soft tissues, including potential pressure or irritation of plantar soft tissue, nerves, or vessels. As a result, the bony outgrowth extending from the first metatarsal head was osteotomised, and wound debridement was performed. Post-operatively, regular dressing was performed. The patient was discharged and advised regular imaging with follow up visits to assess for recurrence of the bony outgrowth or tibial nerve involvement.

## Discussion

Macrodystrophia lipomatosa involves a pathologic overgrowth of adipose tissue within nerves, commonly seen in the median nerve distribution [1]. However, tibial nerve involvement, as presented in our second case, while less frequently documented, has also been reported [2]. The MRI remains the gold standard for diagnosis as it highlights the characteristic admixture of fat and nerve tissue. Typically, T1-weighted images display high signal intensity due to fat, while T2-weighted images can exhibit variable intensities depending on fibrotic components [3].

The FLH is central to MDL’s presentation, often appearing as an enlarged nerve with a “coaxial cable-like” or “spaghetti” configuration-thickened fascicles encased by fatty tissue [4]. This can help differentiate MDL from other entities such as neurofibromatosis or liposarcoma, which may present with similar clinical features but differ considerably in management and prognosis [5]. Although MDL is benign, excessive growth may lead to compressive symptoms, neurovascular compromise, or significant cosmetic concerns [6].

Treatment is guided by symptoms, aesthetic considerations, and functional impairment. Many asymptomatic patients benefit from observation and conservative therapy. Surgical intervention (e.g., partial excision or debulking) may be pursued for symptomatic relief; however, complete excision is typically challenging due to the nerve’s extensive fibrofatty infiltration [7]. In patients with carpal tunnel syndrome secondary to MDL or FLH, carpal tunnel release may provide some symptomatic improvement [8]. Early diagnosis via MRI facilitates more effective treatment planning and may mitigate progressive nerve compression [9].

## Conclusions

These two cases underscore the spectrum of MDL, ranging from more common lower-extremity involvement to the less typical presentation in the median nerve. The MRI findings, especially the presence of diffuse fatty infiltration and nerve enlargement, are pathognomonic for MDL and are paramount to distinguishing it from other potential neuropathies. Timely diagnosis allows for optimized patient care, whether conservative management, surgical intervention, or both. Continued documentation of these rare presentations will enhance our collective understanding and guide better therapeutic strategies.
